# Trends in Periodontitis by Socioeconomic Status in Brazil: National Surveys 1986–2023

**DOI:** 10.1111/jcpe.70130

**Published:** 2026-04-09

**Authors:** Roger Keller Celeste, Erika Augustsson, Rafael Aiello Bomfim, Neda Agahi, Alex Nogueira Haas, Johan Fritzell

**Affiliations:** ^1^ Faculty of Dentistry Federal University of Rio Grande Do Sul Porto Alegre Brazil; ^2^ Aging Research Center Karolinska Institutet & Stockholm University Solna Sweden; ^3^ School of Dentistry Federal University of Mato Grosso Do Sul Campo Grande Brazil

**Keywords:** Brazil, dental health surveys, income, periodontal disease, public health surveillance, socioeconomic factors, time trends

## Abstract

**Aim:**

To describe trends in periodontitis and trends in socioeconomic inequalities in periodontitis in Brazil from 1986 to 2023.

**Methods:**

Representative data were obtained from the SBBrasil surveys of 1986, 2002/2003, 2010 and 2022/2023. Periodontitis was assessed by the community periodontal index (CPI), including clinical attachment loss (CAL) at index teeth. The prevalence of individuals with at least one tooth with periodontal probing depth (PPD) ≥ 4 mm was estimated for 16 capitals and CAL ≥ 4 mm for the whole country according to age group, sex, dental visit in the previous year and household income. Trends were estimated using log‐binomial models, using the sampling design. Interactions were tested by covariates and survey year.

**Results:**

There was a significant decline in the prevalence of periodontitis in all age groups (*p* < 0.01). From 1986 to 2023, the regression‐estimated decline in PPD prevalence during that period was 43% among teenagers and 36% among adults. CAL‐estimated prevalence reduction was 51% from 2003 to 2023. The decline in prevalence was stronger and statistically significant among adults of higher income groups for PPD and CAL, indicating increasing relative inequalities in age groups. Trends did not differ by sex or dental visit.

**Conclusions:**

Periodontal health improved overall, but it was unequally distributed, favouring higher income and younger age groups.

## 1 Introduction

Periodontal diseases encompass chronic inflammatory diseases that are highly prevalent and may lead to tooth loss and poor quality of life. Severe periodontitis is among the most prevalent chronic conditions worldwide and has the second highest burden of oral diseases (Bernabe et al. 2020), affecting more than 20% of individuals aged ≥ 75 years (Nascimento et al. 2024). Moreover, it is projected that severe periodontitis will affect about 1.5 billion people by 2050 because of the growing number of older adults (Nascimento et al. 2024). Nonetheless, periodontal diseases are preventable by addressing modifiable risk factors. These include behaviours such as toothbrushing and smoking, as well as systemic conditions like diabetes and obesity (Genco and Borgnakke 2013; Oppermann et al. 2015). Socioeconomic factors, particularly income and education, are often described as underlying determinants of these conditions and behaviours; therefore, they are associated with periodontal diseases (Albandar 2024; Khajavi et al. 2022). Indeed, there is mounting evidence pointing out that individuals in lower socioeconomic positions have higher rates of periodontal disease (Bastos et al. 2011; Boillot et al. 2001; Borrell and Crawford 2012; Schuch et al. 2017).

There are some studies on trends in periodontitis but all of them were carried out in high‐income countries (HICs). While most have pointed towards a reduction in the prevalence of periodontitis (Borrell et al. 2005; Schürch et al. 2015; Schützhold et al. 2015; Skudutyte‐Rysstad et al. 2007; Wahlin et al. 2018), others have shown stable trends (Edman et al. 2015; Hugoson et al. 2008; Kalsbeek et al. 2000; Li et al. 2021; Rozier et al. 2017) whereas an Australian study reported an increase (Amarasena et al. 2021). Even fewer studies have described such trends by socioeconomic groups, with a weak indication of a reduction in inequalities in periodontitis (Borrell et al. 2005; Li et al. 2021; Rozier et al. 2017). To our knowledge, no trend in the prevalence of periodontitis has been published with data from low‐ and middle‐income countries (LMICs), yet information produced by the Global Burden of Disease study suggests an increase in periodontitis among LMICs and a decline among HICs (Bernabe et al. 2020). This absence of analyses of trends in LMICs means it is unclear whether socioeconomic inequalities are widening, narrowing or remaining stable in settings where the burden of disease is increasing. Noteworthy, trends in periodontitis can be sensitive to changes in diagnostic methods; the use of sub‐optimal and non‐comparable criteria can produce discrepant prevalence estimates ranging from 3% to 49% in the same population and time (Ke et al. 2023).

Monitoring health and socioeconomic inequalities in health is an important surveillance task (Marmot and Goldblatt 2013). Despite several dental surveys in Latin American countries, there appears to be no assessment of trends in the prevalence and inequalities in periodontal diseases (Gjermo et al. 2002; Oppermann et al. 2015). The largest country in the region, Brazil, had its first oral health survey in 1986 with subsequent waves in 2002/2003, 2010 and 2022/2023. During that period in Brazil, there have been improvements in oral hygiene (Christofoli et al. 2021; Gjermo et al. 2002; Oppermann et al. 2015), aligning with trends reported in HICs (Kalsbeek et al. 2000; Raittio et al. 2021; Zaborskis et al. 2023). Additionally, a reduction in the proportion of smokers in all ages and socioeconomic groups (Wendt et al. 2021) may also have improved periodontal health among younger cohorts. Nonetheless, the effect of these behavioural factors may have been offset by an increase in diabetes and obesity, especially among lower socioeconomic groups (Conde et al. 2022; Dos Reis et al. 2022).

Therefore, this study aimed to (i) describe trends in periodontitis in the general population of teenagers and adults and (ii) present trends in socioeconomic inequalities in periodontitis in Brazil from 1986 to 2023.

## 2 Data and Methods

This study is a time trend analysis of four epidemiological dental surveys in Brazil. Data were available for the 1986, 2002/2003, 2010 and 2022/2023 national oral health surveys conducted by the Ministry of Health. The first data collection included a representative sample of urban residents in 16 of the 27 state capitals. The following surveys included a representative sample of the whole country, including the 16 state capitals included in 1986. A full description of the sampling procedures can be obtained at the ‘Brasil Sorridente’ website (https://www.gov.br) and other publications (Roncalli et al. 2012; Vargas et al. 2025). Further information about field work and response rate can be found in Supporting Information (see attached file).

### 2.1 Outcome Variables: Probing Depth and Clinical Attachment Loss

Two outcomes were dichotomous versions of the periodontal assessment based on clinical attachment loss (CAL) and periodontal probing depth (PPD). Individuals with community periodontal index (CPI) scores 3 (PPD 4–5 mm) or 4 (PPD ≥ 6 mm) were considered periodontitis cases. PPD was measured over the four waves in the 16 capitals included in the first survey in 1986 and repeated until 2022/2023. Therefore, trend analyses for PPD were restricted to those capitals. As a sensitivity analysis, we compare the prevalence of periodontitis between the 16 state capitals in the first survey against the full sample for the second, third and fourth surveys (Tables S2 and S3).

Periodontitis was also defined using CAL with a threshold of ≥ 4 mm in at least one index tooth. Although a combination of CAL and PPD has previously been suggested, single indicators have also been used in the literature (Holtfreter et al. 2015) and were adopted in this study. Reasons to analyse CAL and PPD separately are (i) CAL was measured as a categorical variable and this may impute bias when combining with PPD, and (ii) CAL and PPD were not necessarily measured in the same tooth‐site. It was possible to estimate the prevalence of CAL ≥ 4 mm for three waves in 2002/2023, 2010 and 2022/2023 for the whole country.

### 2.2 Income Measures

The first National Oral Health Survey collected data on monthly disposable household income according to the Brazilian Minimum Wage (MW) in three categories (< 3 MW; 3–5 MW; ≥ 5 MW) but no information about the number of individuals in the household was collected in 1986. The second and fourth surveys collected the exact amount of monthly disposable household income from all residents and the number of individuals in the household. The third survey collected the monthly disposable household income in seven categories with an open‐ended upper category; then a continuous income variable was created using the median value for the open‐ended category and the middle value for the closed categories, as previously described (Celeste and Bastos 2013). Further information about the comparability over time can be found in Supporting Information (see attached file), and the sensitivity analyses are presented in Tables S4 and S5, which were used to assess the robustness of socioeconomic gradients observed in the main analyses.

### 2.3 Covariables

Three additional variables were used in the analysis. Age was measured in years and the surveys collected data for the age groups suggested by the World Health Organisation, which were used as a stratification factor: teenagers (aged 15–19 years) and adults (aged 35–44 years). Due to the non‐comparability over time in the oldest age group (aged 50–59 years in 1986 and 65–74 years in subsequent surveys), we only included this in our sensitivity analysis. Sex (males/females) and dental visits (yes/no) were also used as covariables for adjustment. All surveys had a question regarding the time since the last dental visit: ‘How long ago did you go to a dentist?’ and one of the response alternatives was ‘less than a year’; therefore, we were able to re‐categorise different periods into ‘dental visit in the previous year’ (yes/no) and make all surveys comparable.

### 2.4 Statistical Analyses

Descriptive analyses were presented in bivariate tables stratified by age group and survey year using post‐stratification (calibrated) sampling weights provided to estimate weighted prevalence. The first two surveys did not calculate sampling weights; therefore, individuals were assigned a weight of 1. When sampling weights were used, design‐corrected Pearson *χ* ^2^ tests were used to test differences in percentages (using the *svy* command); otherwise, an ordinary *χ* ^2^ test corrected for the clustering of municipalities was used. Sampling weights were not used for regression models because of a lack of convergence with reliable estimates.

Trends were estimated by pooling all survey years using regression models. Adjusted prevalences for each year and income group obtained by age and trends were presented in graphs using the *margins* command after regression analyses. Generalised linear models were fitted using the log‐binomial family with log link and robust variance, adjusting for the clustering of municipalities to estimate prevalence ratios (PRs). Linear trends were tested with year as a continuous variable, and interaction terms were added between year with age groups and income strata. Variables used for adjustment were sex, dental visits and age (within age groups). To avoid a third‐level interaction term, regression models were presented as stratified by age group with two‐level interactions of year and income. The survey year was modelled to predict the total effect over the period. To estimate that, the first year was assigned as 0 and the final survey as 1; this can be interpreted as the total difference from the first to the last year, thus avoiding small yearly changes when using the calendar year. Then, for the PPD analysis this was 1986 = 0, 2003 = 0.46, 2010 = 0.65 and 2023 = 1; and for CAL analysis this was 2003 = 0, 2010 = 0.35 and 2023 = 1.

## 3 Results

The eligible sample size in state capitals was 25,638 individuals with data for the 1986, 2003, 2010 and 2023 surveys used for PPD analysis. Since 934 individuals had no teeth, an additional 679 were with missing index teeth and a further 258 had missing values on income, the analytical sample size for PPD was 23,767 (Tables 1 and S1 for details by age, sex, income and dental visits). There were no missing values for age and sex, but for dental visits it varied between 1.2% and 4.6%, and for income, it was up to 30% in 2023 (Table S1).

**TABLE 1:** Number of cases and unadjusted/unweighted prevalence of two periodontal outcomes by survey year and age group in Brazil.

Survey year	Total sample size	Cases	Prevalence	(95% CI)
Community periodontal index; periodontal probing depth ≥ 4 mm				
Teenagers 15–19 years				
1986	4731	368	7.8%	(5.4–11.1)
2002/2003	1135	14	1.2%	(0.5–2.8)
2010	2355	188	8.0%	(5.3–11.9)
2022/2023	3815	170	4.5%	(2.9–6.8)
Adults 35–44 years				
1986	2262	631	27.9%	(22.2–34.5)
2002/2003	973	117	12.0%	(7.9–17.8)
2010	4227	1181	27.9%	(24.0–32.3)
2022/2023	4269	719	16.8%	(13.9–20.2)
Community periodontal index–clinical attachment loss ≥ 4 mm				
Adults 35–44 years				
2002/2003	12,206	4081	33.4%	(31.0–36.0)
2010	9506	2558	26.9%	(24.0–30.0)
2022/2023	8867	1463	16.5%	(15.0–18.1)

*Note:* Periodontal analysis for periodontal probing depth sampling included the same 16 state capitals selected in the first wave in 1986. Analysis for clinical attachment loss included a random representative sample of Brazilian cities since 2002/2003.

The sample size for the whole country was 32,229 individuals with data for the 2003, 2010 and 2023 surveys used for CAL analysis. Since 1616 individuals had no teeth and further 34 had missing index teeth, the analytical sample size for CAL was 30,579 (Tables 1 and S1 for details by age, sex, income and dental visits). There were no missing values for age and sex, but for dental visits it varied between 1.7% and 5.4% and for income it was up to 29% in 2023 (Table S1).

Sociodemographic variables remained relatively stable. Considering the whole country (2003–2023 samples), there was a consistent proportion of women every year, around 60%, and the proportion of households with < 3 MW monthly was about 44% and 51% in 2003 and 2023, respectively. The proportion of individuals visiting the dentist increased from 39% to 46% between 2003 and 2023.

### 3.1 Trends in Periodontal Probing Depth

There was a general decline in the prevalence of periodontitis, defined as PPD ≥ 4 mm in all age groups (Tables 1 and 2). Among teenagers, the unweighted prevalence reduced from 7.8% in 1986 to 4.5% in 2023, with a regression‐based decline of approximately 43% over 37 years (see Table 3, adjusted prevalence ratio (PR) = 0.57, 95% confidence interval [95% CI]: 0.37–0.89). Among adults, the unweighted prevalence reduced from 27.9% in 1986 to 16.8% in 2023, with a regression‐based decline of 36% over 37 years (see Table 3, PR = 0.64, 95% CI: 0.50–0.81).

**TABLE 2:** Weighted prevalence (%) of community periodontal index ≥ 4 of periodontal probing depth according to income level and survey year in 16 Brazilian capitals.

	Survey year	1986			2002/2003			2010			2022/2023		
		%	[95% CI]	*p*	%	[95% CI]	*p*	%	[95% CI]	*P*	%	[95% CI]	*p*
Young 15–19 years													
Total		7.8	[5.4, 11.1]		1.2	[0.5, 2.8]		8.9	[5.1, 14.9]		5.9	[3.5, 9.6]	
Total household income	< 3 MW	11.2	[8.9, 14.1]	< 0.01	2.1	[1.0, 4.5]	0.03	8.3	[5.0, 13.6]	0.20	6.7	[4.2, 10.6]	0.02
	3–5 MW	7.5	[4.8, 11.7]		0.4	[0.0, 3.7]		11.2	[5.0, 23.3]		4.1	[1.9, 8.6]	
	≥ 5 MW	4.9	[2.5, 9.2]		0.3	[0.0, 2.7]		3.3	[1.1, 9.4]		1.4	[0.4, 4.6]	
Sex	Males	7.3	[4.9, 10.7]	0.30	1.2	[0.3, 4.7]	0.98	6.7	[4.5, 9.9]	0.10	4.8	[2.9, 7.9]	< 0.01
	Females	8.1	[5.5, 11.6]		1.2	[0.6, 2.6]		10.7	[5.2, 20.7]		6.1	[3.8, 9.6]	
Visited the dentist last year?	No	9.8	[6.7, 14.2]	0.03	2.0	[0.8, 5.0]	0.05	11.6	[5.6, 22.4]	0.03	5.9	[3.9, 9.0]	0.02
	Yes	6.7	[4.5, 9.9]		0.6	[0.2, 1.9]		6.1	[4.5, 8.1]		4.2	[2.7, 6.7]	
Adults 35–44 years													
Total		27.9	[22.2, 34.5]		12.0	[7.9, 17.8]		27.5	[23.1, 32.3]		16.8	[14.9, 18.8]	
Total household income	< 3 MW	29.2	[23.0, 36.1]	0.49	15.0	[9.7, 22.5]	0.06	31.0	[24.7, 38.0]	< 0.01	19.5	[17.0, 22.3]	0.04
	3–5 MW	27.4	[20.4, 35.6]		12.2	[6.8, 20.9]		24.1	[20.7, 27.9]		15.4	[12.2, 19.2]	
	≥ 5 MW	26.4	[20.4, 33.3]		8.0	[4.0, 15.1]		11.5	[8.5, 15.5]		9.4	[4.6, 18.2]	
Sex	Males	30.7	[25.3, 36.7]	0.03	17.6	[10.6, 27.9]	0.01	30.0	[23.7, 37.2]	0.04	18.1	[15.8, 20.6]	0.11
	Females	26.9	[20.9, 33.9]		9.3	[6.1, 14.0]		26.4	[22.4, 30.7]		16.1	[14.0, 18.4]	
Visited the dentist last year?	No	30.3	[23.4, 38.2]	0.13	11.4	[7.6, 16.9]	0.58	32.1	[27.1, 37.5]	< 0.01	19.1	[16.5, 22.1]	0.01
	Yes	25.3	[19.3, 32.5]		12.9	[7.7, 20.9]		23.0	[18.8, 27.8]		15.0	[12.7, 17.7]	

**TABLE 3:** Adjusted prevalence ratio (PR) and with 95% confidence interval (95% CI) of having periodontal probing depth ≥ 4 mm according to covariates in log‐binomial regression over 1986–2023.

Variable	Category	Crude PR	95% CI	Adj. PR	95% CI
Teenagers (15–19 years)					
Main effects					
Sex	Males	1		1	
	Females	1.00	(0.87–1.15)	1.00	(0.87–1.15)
Age	Continuous (years)	1.15	(1.08–1.23)	1.16	(1.08–1.23)
Have you visited the dentist last year?	No	1		1	
	Yes	0.80	(0.69–0.93)	0.79	(0.68–0.92)
Household income group in minimum wages (MW)	< 3 MW	1		1	
	3–5 MW	0.76	(0.65–0.89)	0.71	(0.55–0.92)
	≥ 5 MW	0.47	(0.30–0.71)	0.52	(0.30–0.90)
Survey year	Continuous (37 years difference)	0.57	(0.36–0.89)	0.57	(0.37–0.87)
Two‐way interaction					
Income × survey year	3–5 MW × years			1.31	(0.65–2.61)
	≥ 5 MV × years			0.35	(0.08–1.64)
Adults 35–44 years					
Main effects					
Sex	Males	1		1	
	Females	0.82	(0.74–0.90)	0.81	(0.74–0.90)
Age	Continuous (years)	1.02	(1.01–1.03)	1.02	(1.01–1.03)
Have you visited the dentist last Year?	No	1		1	
	Yes	0.84	(0.76–0.94)	0.84	(0.76–0.93)
Household income group in minimum wages (MW)	< 3 MW	1		1	
	3–5 MW	0.85	(0.73–0.98)	0.94	(0.78–1.13)
	≥ 5 MW	0.58	(0.46–0.74)	0.86	(0.71–1.04)
Survey year	Continuous (37 years difference)	0.64	(0.50–0.81)	0.74	(0.59–0.93)
Two‐way interaction					
Income × Survey year	3–5 MW × years			0.84	(0.57–1.24)
	≥ 5 MV × years			0.32	(0.20–0.53)

*Note:* The interaction between income and survey year was *p* = 0.21 among teenagers and *p* < 0.01 among adults.

**FIGURE 1:**
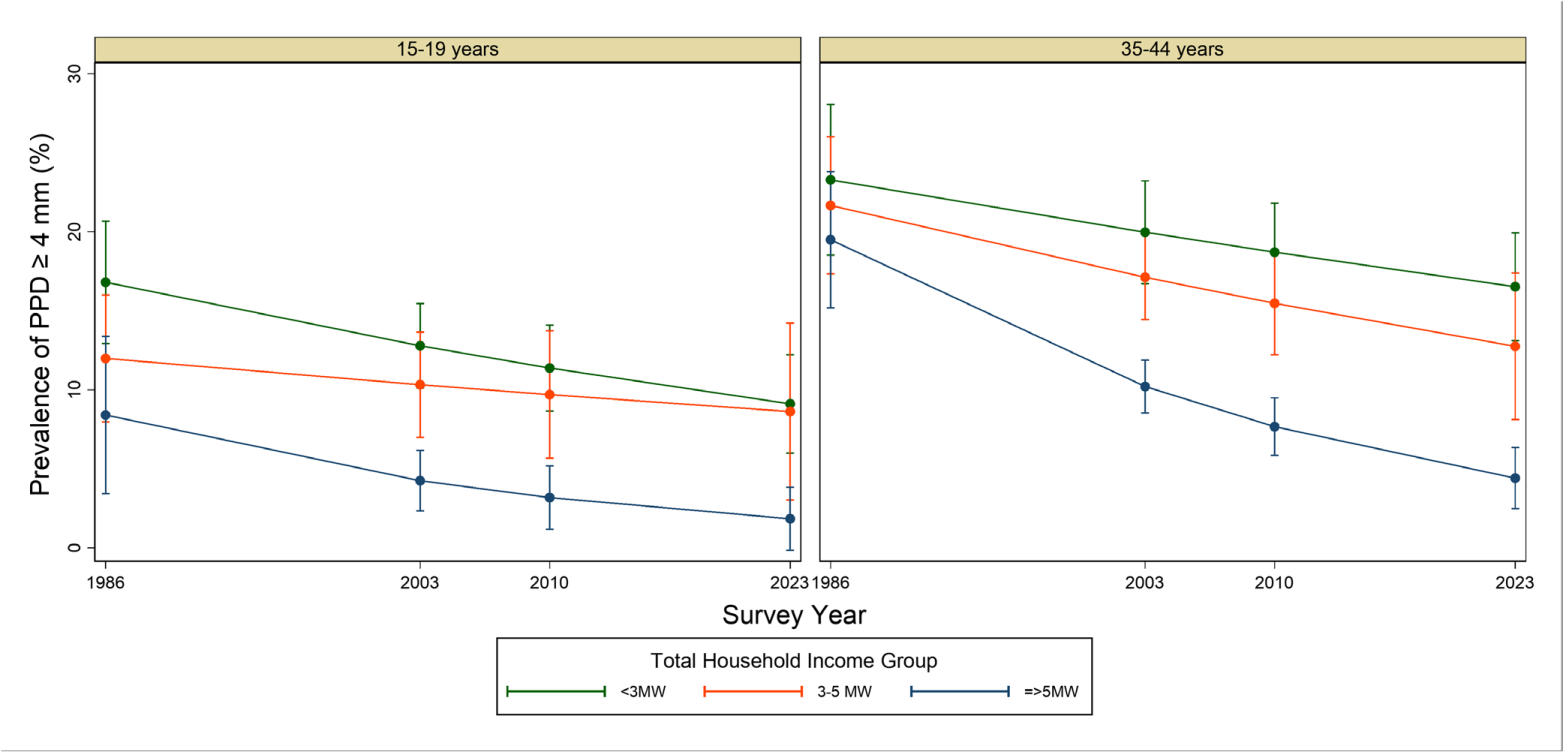
Trends in prevalence and 95% confidence intervals (for each survey year) of community periodontal index scores 4 and 5 (periodontal probing depth ≥ 4 mm) in three income groups in 16 Brazilian state capitals between 1986 and 2023 (Test of differences of trends among income groups: (a) 15–19 year *p* = 0.21; (b) 35–44 years *p* < 0.01).

There was a consistent income gradient, with lower income groups having higher prevalence in almost all age brackets and survey years (Table 2). The relative inequalities in PPD have increased among adults, as the decline was larger for those with higher income (Table 3 and Figure 1). Interaction terms between income and survey year were statistically significant among adults (*p* < 0.01) but not among teenagers (*p* = 0.21).

Multivariable regression results are presented in Table 3. In adjusted analysis, women consistently presented lower prevalence ratios of having PPD ≥ 4 mm, although not statistically significant (*p* > 0.05) among the teenagers. Having visited the dentist in the previous years was also associated with a lower prevalence of PPD ≥ 4 mm among all age groups.

### 3.2 Trends in Clinical Attachment Loss

There was a general decline in the prevalence of CAL ≥ 4 mm among adults (Tables 1 and 4). The unweighted prevalence reduced from 33.4% in 2003 to 18.2% in 2023, with an estimated regression‐based decline of 51% over 20 years (see Table 5, adjusted PR = 0.49 [95% CI: 0.43–0.56]).

**TABLE 4:** Weighted prevalence (%) of clinical attachment loss ≥ 4 mm by survey year among adults in Brazil.

		2002/2003			2010			2022/2023		
		%	[95% CI]	*p*	%	[95% CI]	*p*	%	[95% CI]	*p*
Adults 35–44 years										
Total		33.4	[31.0, 36.0]		29.1	[23.9, 34.8]		18.2	[15.1, 21.7]	
Total household income	< 3 MW	35.8	[33.1, 38.7]	< 0.01	31.1	[25.4, 37.5]	0.01	19.5	[15.3, 24.4]	0.47
	3–5 MW	31.9	[28.7, 35.3]		25.5	[20.6, 31.1]		18.9	[13.3, 26.2]	
	≥ 5 MW	27.1	[23.5, 31.0]		21.1	[13.3, 31.8]		13.0	[6.0, 25.9]	
Sex	Males	39.8	[36.7, 42.9]	< 0.01	32.1	[27.3, 37.4]	0.06	20.4	[16.1, 25.6]	0.09
	Females	30.2	[27.7, 32.8]		27.2	[21.3, 34.2]		17.0	[14.0, 20.6]	
Visited the dentist last year?	No	35.4	[32.7, 38.1]	< 0.01	28.7	[23.4, 34.7]	0.81	23.5	[16.9, 31.7]	0.01
	Yes	30.5	[27.9, 33.3]		29.5	[23.0, 37.0]		13.3	[10.7, 16.4]	

**TABLE 5:** Adjusted prevalence ratio (PR) and with 95% confidence interval (95% CI) of having clinical attachment loss ≥ 4 mm according to covariates in log‐binomial regression over 2002–2023.

Variable	Category	PR	(95% CI)	PR	(95% CI)
Adults (35–44 years)					
Main effects					
Sex	Males	1		1	
	Females	0.79	(0.75–0.83)	0.78	(0.75–0.82)
Age	Continuous (years)	1.06	(1.05–1.06)	1.06	(1.05–1.06)
Have you visited the dentist last Year?	No	1		1	
	Yes	0.91	(0.87–0.95)	0.91	(0.87–0.95)
Household Income group in minimum wages (MW)	< 3 MW	1		1	
	3–5 MW	0.84	(0.79–0.90)	0.87	(0.80–0.94)
	≥ 5 MW	0.71	(0.63–0.80)	0.75	(0.66–0.86)
Survey year	Continuous (20 years difference)	0.49	(0.43–0.56)	0.52	(0.45–0.59)
Two‐way interaction					
Income × Survey year	3–5 MW × years			0.86	(0.69–1.07)
	≥ 5 MV × years			0.71	(0.53–0.94)

*Note:* The interaction between income and survey year was statistically significant (*p* = 0.04).

**FIGURE 2:**
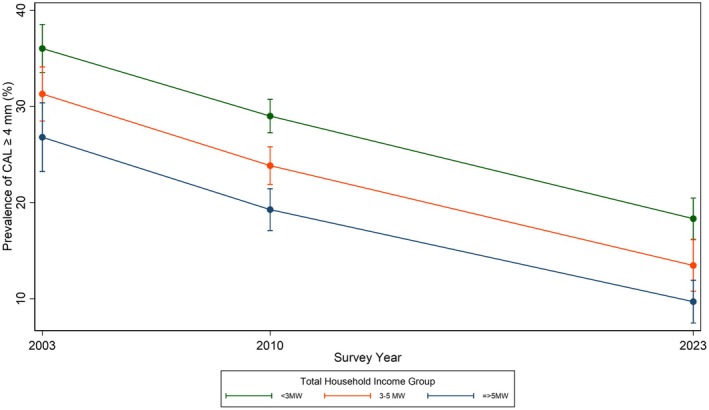
Trends in prevalence and 95% confidence intervals (for each survey year) of clinical attachment loss ≥ 4 mm in three income groups in Brazil between 2003 and 2023 (Test of differences of trends among income groups, *p* = 0.04).

Among the adults, there was an income gradient, with lower income groups having higher prevalence (Table 4). The relative income inequalities have increased, as the decline was more among those with higher income (Table 5 and Figure 2). Interaction terms between income and survey year were statistically significant among adults (*p* < 0.01).

Multivariable regression results are presented in Table 5. In adjusted analysis, women consistently presented lower prevalence ratios of having CAL ≥ 4 mm. Having visited the dentist in the previous years was also associated with lower prevalence of CAL ≥ 4 mm.

### 3.3 Sensitivity Analysis

Trends concerning older adults were only presented as supporting data (Tables S1–S5; Figures S1 and S2) because missing information was very high due to edentulism (45.1% in 1986 and 32.1% in 2023 among state capitals and 54.3% in 2002 and 38.8% in 2023 in the whole country). Additionally, the age group in 1986 (50–59 years) did not overlap with subsequent waves (65–74 years). Finally, we present different ways to estimate prevalence, using weighted and unweighted estimates in combination with age and sex standardisation (Table S6), but such results did not differ significantly for teenagers and adults.

There was a decline in the prevalence of PPD ≥ 4 mm but not CAL ≥ 4 mm among older adults. The estimated regression‐based prevalence decline, adjusted for age, sex and dental visits, was 46% over 37 years for PPD (PR = 0.54; 95% CI: 0.38–0.77) and 9% over 20 years for CAL (PR = 0.91; 95% CI: 0.79–1.03). The trends differed among income groups for PPD (Figure S1, *p* = 0.01) but not for CAL > 4 mm (Figure S2, *p* = 0.96). Further information about additional interaction results can be found in Supporting Information (see attached file).

## 4 Discussion

This study describes the trends in periodontitis in Brazil up to 37 years and whether socioeconomic inequalities in periodontitis have changed during this period. First, confirming reports from HICs, we noticed a general decrease in adjusted prevalence of CAL up to 51% reduction among 35–44‐year‐olds and up to 43% in PPD among 15–19‐year‐olds. Importantly, this is the first known study in Latin America and is likely the first among LMICs to address this topic. Second, relative inequalities in PPD and CAL are large and seem to be increasing because the higher income group showed most of the improvements.

A general decline in periodontitis appears robust, although it is not strictly linear for PPD because the 2003 survey presented atypically lower values. Previous studies suggested a reduction in the prevalence in periodontal diseases among HICs (Borrell et al. 2005; Schürch et al. 2015; Schützhold et al. 2015; Skudutyte‐Rysstad et al. 2007; Wahlin et al. 2018). Large variability in prevalence has been reported because of several issues, which could partially explain the fluctuations over time (Ke et al. 2023). The present work did not aim to explain the reasons for declining prevalence, but in Brazil, they may be related to improvements in risk factors such as the reduction in smoking and improvements in oral hygiene. The improvement in the former is well described (Wendt et al. 2021), and despite possible measurement concerns, trends in oral hygiene also seem to be improving over the past decades (Christofoli et al. 2021; Oppermann et al. 2015). For example, a recent study reported an increase in the use of toothbrushes, toothpaste and dental floss from 52% to 62% between 2013 and 2019 and a reduction in socioeconomic differences (Teixeira and Souza Júnior 2025). It is possible that the effect of the increase in the prevalence of diabetes and obesity (Conde et al. 2022; Dos Reis et al. 2022; Ferreira et al. 2021) will be seen in future cohorts.

Despite overall declines in periodontitis, socioeconomic inequalities have persisted. Improvements in PPD disproportionately favoured higher income groups, indicating widening absolute and relative disparities, whereas inequalities in CAL remained stable but still favoured the affluent. These patterns underscore the complex dynamics between population health improvements and social equity. A decline in prevalence with maintenance of inequalities has also been reported among HICs (Borrell et al. 2005; Rozier et al. 2017; Li et al. 2021). Explaining trends in inequalities can be challenging, and it has been suggested that it may be a result of differential exposure or differential effects of risk factors (Diderichsen et al. 2019), but also the effect called the inverse equity hypothesis (Victora et al. 2000). Similar trends have been described for tooth loss among teenagers and adults in Brazil (Celeste et al. 2011; Roncalli et al. 2015). The diverging trend by socioeconomic group may be attributable to changes in the proportion of a mix of different risk factors across social strata. It is, however, theoretically possible that disadvantaged groups may have fewer resources and abilities to recover or cope with strong hazards and develop the disease to a larger extent—even being equally exposed—a concept called differential susceptibility (Diderichsen et al. 2019). For example, healing from periodontal treatment is faster among individuals who do not smoke and do not have diabetes—usually the richer ones. Such a hypothesis needs to be further investigated with appropriate information, particularly in longitudinal studies.

## 5 Strengths and Limitations

A major novelty of this study is the presentation of data on how trends and socioeconomic inequalities of periodontal disease look like in a non‐HIC setting. Further, we were able to show a long‐term trend with comparable diagnostic criteria. Sample sizes were large and representative, either for the whole country (CAL) or 16 capitals (PPD), and thus had sufficient statistical power to assess trends, despite a declining response rate over time, a lower sample size in 2003 and high missingness for the income variable in 2023. Importantly, the two first waves did not report non‐response and did not have sampling weights, which might have compromised representativeness. In this study, economic measures were adjusted for inflation, allowing for comparisons across time by socioeconomic groups stratified by age, sex and dental visits. On the other hand, there are limitations. The well‐known limitations of CPI should be acknowledged. Moreover, the possible bias on the assessment of periodontal variables should not be discarded because full data for examiners' reproducibility were not provided in the full reports of survey waves. Also, the trends in PPD were not representative for the whole country and were restricted to state capitals. However, the main objective of this study was to present trends, and we used the best data currently available. Our trend analysis of periodontitis was hampered by the fact that there were only four or three measurement points, and a clear linear trend would not be expected. The economic measure used had only three categories, and the gradient was not smooth in some subgroups. Indeed, total household income poverty estimates are significantly lower than per capita or equalised income, but it has a small effect on associations which are slightly stronger (Celeste and Bastos 2013). The use of education may be an alternative that would have excluded data from 1986. Finally, we were not able to investigate potential explanations for such trends.

## 6 Conclusions and Policy Implication

These findings underscore the need to strengthen implementation strategies within oral health policies to ensure equitable improvements in periodontal health. The unequal gains observed suggest that the current public dental service coverage in primary care—approximately 30% of the total population (Pilotto and Celeste 2021)—has not provided sufficient benefits to those most in need. To address these challenges, strategies could include integrating periodontal management into care pathways for chronic diseases that disproportionately affect lower income populations (such as diabetes and obesity). Oral health programmes need to combine individual approaches with clinical adaptations that consider general health status and physical limitations, with community interventions integrated into primary health care (Chan et al. 2025). Additionally, strengthening the surveillance of periodontal conditions and related risk factors within health information systems could better inform targeted local interventions with a growing population, in line with international guidelines for healthy ageing (World Health Organization 2024).

Despite an overall improvement in periodontal health, there is still a persistent and increasing socioeconomic gap. Future studies should investigate potential explanations for trends that have now been identified, considering that the current findings are compatible with period and cohort effect. A decline in prevalence among older cohorts might be seen in future cohorts, but the effect of an increase in the number of teeth per individual should be studied, as this may hide a more positive outcome. Policymakers and clinicians should concentrate their efforts on controlling risk factors that are unevenly distributed in the population to improve overall oral health and decrease inequalities.

## Author Contributions

All authors contributed to the conception of the study. R.K.C. wrote the initial draft and carried out analyses. All authors participated in the interpretation of results, critically reviewed and edited the manuscript and approved the final version.

## Funding

This work was supported by Forskningsrådet om Hälsa, Arbetsliv och Välfärd (2023‐01595).

## Conflicts of Interest

The authors declare no conflicts of interest.

## Supporting information


**Data S1:** Supporting Information.

**Table S1:** Percentage of individuals according to socioeconomic and demographic variables by survey year in a representative sample and state capitals.
**Table S2:** Weighted prevalence of community periodontal index scores by age group and survey year in 16 Brazilian capitals.
**Table S3:** Weighted prevalence of clinical attachment loss categories by survey year and age group in Brazil.
**Table S4:** Weighted prevalence of pocket depth ≥ 4 mm (community periodontal index scores 4 and 5) according to income by age group and survey year in representative samples of Brazilians.
**Table S5:** Weighted prevalence of clinical attachment loss ≥ 4 mm according to income by age group and survey year in representative samples of Brazilians.
**Table S6:** Sensitivity analysis of standardised populations.
**Figure S1:** Trends in prevalence and 95% confidence intervals (for survey years) of community periodontal index scores 4 and 5 (periodontal probing depth ≥ 4 mm) adjusted by sex, age and dental visits in three income groups in Brazilian state capitals between 1986 and 2023 (Test of differences of trends among income groups: (a) 15–19 years *p* = 0.21; (b) 35–44 years *p* < 0.01; (c) 50–74 years *p* = 0.01).
**Figure S2:** Trends in prevalence and 95% confidence intervals (for survey years) of clinical attachment loss ≥ 4 mm adjusted by sex, age and dental visits in three income groups in Brazil between 2003 and 2023 (Test of differences of trends among income groups: (a) 35–44 years *p* = 0.04; (b) 65–74 years *p* = 0.96).

## Data Availability

The data that support the findings of this study are openly available in Ministery of Health of Brazil at https://www.gov.br/saude/pt‐br/composicao/saps/brasil‐sorridente/sb‐brasil/dados.
