# Supplementary material for: Trends in Periodontitis by Socioeconomic Status in Brazil: National Surveys 1986–2023
Source: J Clin Periodontol. 2026 Apr 9;53(6):901–10. doi: 10.1111/jcpe.70130 (PMC13167646; doi:10.1111/jcpe.70130)
Supplement: Supplementary file 1 — Data S1: Supporting Information. [file JCPE-53-901-s001.docx]

**Supplementary Information**

# **Data and Methods**

Adults were interviewed using a structured questionnaire and were clinically examined by a dentist in their homes. The response rate was not available for 1986; however, in 2002/2003, it was reported that 85% of the planned sample size was achieved. In 2010, the overall household response rate was 77.9% among the 15-19-year-olds and 61.1% among 35-44-year-olds. In 2023, the household response rate varied between states from 21.6% (Espirito Santo) to 99.7% (Sergipe) for both age groups.

The field work procedures followed the recommendations of the World Health Organization (WHO) guidelines. Diagnostic agreement between examiners was evaluated using weighted kappa statistics. Specific information for the calibration procedures in each survey was not provided by the Ministry of Health in their publications. It was reported that a kappa value of 0.65 for 2002/2003 and 2010, and of 0.61 for 2022/2023 were the lowest acceptable value for the conduction of the field work.

## Outcome Variables: Probing Depth and Clinical Attachment Loss

The same examination protocol for population surveys idealized by the World Health Organization (WHO) was applied in all surveys. Therefore, the Community Periodontal Index (CPI) was used in all surveys for individuals aged 15 or over, and it included information about bleeding on probing, dental calculus, shallow (4–5 mm) and deep (≥ 6 mm) pocket depths. Data on CAL was only collected in the three last surveys, and only for individuals aged 35 or over according to the following categories: up to 3 mm, 4–5 mm, 6–8 mm, 9–11 mm, and > 12 mm, also in index teeth. Six index teeth per person were examined in different tooth sites and the highest score was used to represent each sextant. A sextant was excluded if less than two teeth were present or, for attachment loss it was also excluded if it was not possible to examine the index teeth due to calculus or other reasons. The classic WHO ball-ended periodontal probe was used in all surveys.

## Income Measures

For the primary analyses, income was categorised into three MW-based groups consistent with the 1986 survey and interpreted as a relative socioeconomic ranking within each survey wave, rather than as a measure of absolute purchasing power. This approach allows long-term trend analyses while avoiding artificial harmonisation across historically and methodologically distinct surveys. Brazilian MW is expected to keep purchasing power and is corrected for inflation yearly by the government based on the National Consumer Price Index (INCP in Portuguese); it is a relative socioeconomic ranking within each survey wave rather than an absolute measure of income. Converted to US dollars, the Brazilian MW was approximately in 1986, 2003, 2010 and 2023: ~58US$, 63US$, 297US$, 267US$.

Because household size information was unavailable in 1986, income equivalization was not possible across all waves. Therefore, equivalized household income, defined as total household income divided by the square root of the number of household members, was used in sensitivity analyses restricted to the 2002/2003, 2010, and 2022/2023 surveys. Results from these analyses are presented in Supplementary Tables S4 and S5 and were used to assess the robustness of socioeconomic gradients observed in the main analyses.

# **Results**

## Sensitivity Analysis

We tested interaction terms to assess if the decline in CAL was different between women and men (p=0.54 for adult and p=0.39 for older adults) and those who visited and did not visit the dentist in the previous years (p=0.10 for adults and p=0.40 for older adults). A similar pattern was observed when we tested interaction terms to assess if the decline in PPD was different between women and men (p=0.89 for teenagers, p=0.38 for adult and p<0.01 for older adults) and those who visited and did not visit the dentist in the previous years (p=0.47 for teenagers, p=0.77 for adults and p=0.28 for older adults).
