# Supplementary material for: Trends in Periodontitis by Socioeconomic Status in Brazil: National Surveys 1986–2023
Source: J Clin Periodontol. 2026 Apr 9;53(6):901–10. doi: 10.1111/jcpe.70130 (PMC13167646; doi:10.1111/jcpe.70130)
Supplement: Supplementary file 2 — Table S1: Percentage of individuals according to socioeconomic and demographic variables by survey year in a representative sample and state capitals. Table S2: Weighted prevalence of community periodontal index scores by age group and survey year in 16 Brazilian capitals. Table S3: Weighted prevalence of clinical attachment loss categories by survey year and age group in Brazil. Table S4: Weighted prevalence of pocket depth ≥ 4 mm (community periodontal index scores 4 and 5) according to income by age group and survey year in representative samples of Brazilians. Table S5: Weighted prevalence of clinical attachment loss ≥ 4 mm according to income by age group and survey year in representative samples of Brazilians. Table S6: Sensitivity analysis of standardised populations. Figure S1: Trends in prevalence and 95% confidence intervals (for survey years) of community periodontal index scores 4 and 5 (periodontal probing depth ≥ 4 mm) adjusted by sex, age and dental visits in three income groups in Brazilian state capitals between 1986 and 2023 (Test of differences of trends among income groups: (a) 15–19 years p = 0.21; (b) 35–44 years p < 0.01; (c) 50–74 years p = 0.01). Figure S2: Trends in prevalence and 95% confidence intervals (for survey years) of clinical attachment loss ≥ 4 mm adjusted by sex, age and dental visits in three income groups in Brazil between 2003 and 2023 (Test of differences of trends among income groups: (a) 35–44 years p = 0.04; (b) 65–74 years p = 0.96). [file JCPE-53-901-s002.docx]

Table S1 – Percentage of individuals according to socioeconomic and demographic variables by survey year in a representative sample and state capitals.

|  |  | 1986 | | 2002/2003 | | 2010 | | 2022/2023 | |
| --- | --- | --- | --- | --- | --- | --- | --- | --- | --- |
|  |  | Sample size (n) | % | Sample size (n) | % | Sample size (n) | % | Sample size (n) | % |
| Representative Sample | | | | | | | | | |
| Total |  |  |  | 35613 | 100.0 | 22843 | 100.0 | 26808 | 100.0 |
| Clinical Attachment Loss >=4mm | No teeth |  |  | 12591 | 35.4 | 12230 | 53.5 | 13780 | 51.4 |
|  | 1+ teeth |  |  | 2062 | 5.8 | 1303 | 5.7 | 904 | 3.4 |
|  | Missing |  |  | 20960 | 58.9 | 9310 | 40.8 | 12124 | 45.2 |
| Age groups | 15-19 years |  |  | 16832 | 47.3 | 5445 | 23.8 | 8053 | 30.0 |
|  | 35-44 years |  |  | 13431 | 37.7 | 9779 | 42.8 | 9019 | 33.6 |
|  | 65-74 years |  |  | 5350 | 15.0 | 7619 | 33.4 | 9736 | 36.3 |
| Sex | Males |  |  | 13442 | 37.7 | 8774 | 38.4 | 10458 | 39.0 |
|  | Females |  |  | 22171 | 62.3 | 14069 | 61.6 | 16350 | 61.0 |
| Have you visited the Dentist last Year? | No |  |  | 21033 | 59.1 | 13108 | 57.4 | 13666 | 51.0 |
|  | Yes |  |  | 13991 | 39.3 | 9289 | 40.7 | 11706 | 43.7 |
|  | Missing |  |  | 589 | 1.7 | 446 | 2.0 | 1436 | 5.4 |
| Equivalent Household Income | <=1/2MW |  |  | 7846 | 22.0 | 2514 | 11.0 | 2956 | 11.0 |
|  | 1/2-1MW |  |  | 10234 | 28.7 | 7319 | 32.0 | 6753 | 25.2 |
|  | 1-2MW |  |  | 10309 | 29.0 | 7339 | 32.1 | 6102 | 22.8 |
|  | =>2MW |  |  | 6777 | 19.0 | 4824 | 21.1 | 3003 | 11.2 |
|  | Missing |  |  | 447 | 1.3 | 847 | 3.7 | 7994 | 29.8 |
| State Capitals | | | | | | | | | |
|  |  | Sample size (n) | % | Sample size (n) | % | Sample size (n) | % | Sample size (n) | % |
| Total |  | 10366 | 100.0 | 2508 | 100.0 | 10493 | 100.0 | 12563 | 100.0 |
| Pocket depth >=4mm | No teeth | 6404 | 61.8 | 2126 | 84.8 | 6601 | 62.9 | 9367 | 74.6 |
|  | 1+ teeth | 1246 | 12.0 | 151 | 6.0 | 2037 | 19.4 | 1465 | 11.7 |
|  | Missing/Excluded | 2716 | 26.2 | 231 | 9.2 | 1855 | 17.7 | 1731 | 13.8 |
| Age groups | 15-19 years | 4776 | 46.1 | 1138 | 45.4 | 2418 | 23.0 | 3845 | 30.6 |
|  | 35-44 years | 3336 | 32.2 | 1022 | 40.8 | 4410 | 42.0 | 4355 | 34.7 |
|  | 50-74 years | 2254 | 21.7 | 348 | 13.9 | 3665 | 34.9 | 4363 | 34.7 |
| Sex | Males | 3206 | 30.9 | 930 | 37.1 | 3860 | 36.8 | 4798 | 38.2 |
|  | Females | 7160 | 69.1 | 1578 | 62.9 | 6633 | 63.2 | 7765 | 61.8 |
| Have you visited the Dentist last Year? | No | 4836 | 46.7 | 1327 | 52.9 | 5677 | 54.1 | 6339 | 50.5 |
|  | Yes | 5405 | 52.1 | 1091 | 43.5 | 4649 | 44.3 | 5646 | 44.9 |
|  | Missing | 125 | 1.2 | 90 | 3.6 | 167 | 1.6 | 578 | 4.6 |
| Income group in Minimum Wages (MW) | <3MW | 4262 | 41.1 | 1181 | 47.1 | 6243 | 59.5 | 6466 | 51.5 |
|  | 3-5 MW | 2934 | 28.3 | 502 | 20.0 | 3112 | 29.7 | 1373 | 10.9 |
|  | =>5 MW | 3170 | 30.6 | 764 | 30.5 | 690 | 6.6 | 912 | 7.3 |
|  | Missing | 0 | 0.0 | 61 | 2.4 | 448 | 4.3 | 3812 | 30.3 |

Table S2 – Weighted prevalence of Community Periodontal Index scores by age group and survey year in 16 Brazilian capitals.

|  |  | Community Periodontal Index | | | | | |
| --- | --- | --- | --- | --- | --- | --- | --- |
| Survey Year |  | Sound | Bleeding | Calculus | Pocket 4-5mm | Pocket 6+ mm | Total |
| Young 15-19 years | | | | | | | |
| 1986 (capitals) | Weighted Prevalence | **30.1** | **20.1** | **42.0** | **7.1** | **0.7** | **100** |
|  | [95% CI] | [22.5,39.0] | [14.3,27.4] | [32.8,51.9] | [5.1,9.8] | [0.3,1.6] |  |
| 2002/2003 (capitals) | Weighted Prevalence | **47.2** | **19.4** | **32.2** | **1.0** | **0.3** | **100** |
|  | [95% CI] | [36.5,58.2] | [14.7,25.2] | [22.1,44.3] | [0.4,2.3] | [0.1,0.9] |  |
| 2002/2003 (all sample) | Weighted Prevalence | **46.3** | **18.8** | **33.5** | **1.2** | **0.2** | **100** |
|  | [95% CI] | [43.4,49.3] | [17.4,20.4] | [30.7,36.4] | [0.9,1.6] | [0.1,0.2] |  |
| 2010 (capitals) | Weighted Prevalence | **43.0** | **9.0** | **39.1** | **8.1** | **0.8** | **100** |
|  | [95% CI] | [30.0,57.1] | [7.5,10.7] | [30.1,48.9] | [4.9,13.0] | [0.3,2.5] |  |
| 2010 (all sample) | Weighted Prevalence | **51.3** | **10.0** | **28.7** | **9.3** | **0.8** | **100** |
|  | [95% CI] | [44.8,57.8] | [7.6,12.9] | [23.8,34.0] | [7.1,12.0] | [0.4,1.4] |  |
| 2022/2023 (capitals) | Weighted Prevalence | **49.6** | **11.0** | **33.9** | **5.1** | **0.4** | **100** |
|  | [95% CI] | [44.6,54.7] | [9.2,13.1] | [30.4,37.6] | [3.2,8.0] | [0.2,0.9] |  |
| 2022/2023 (all sample) | Weighted Prevalence | **53.5** | **9.8** | **31.9** | **4.4** | **0.3** | **100** |
|  | [95% CI] | [49.4,57.5] | [8.2,11.8] | [28.2,35.9] | [3.1,6.2] | [0.2,0.6] |  |
| Adults 35-44 years | | | | | | | |
| 1986 (capitals) | Weighted Prevalence | **11.4** | **12.4** | **48.3** | **21.3** | **6.6** | **100** |
|  | [95% CI] | [6.9,18.3] | [8.2,18.4] | [40.8,55.8] | [17.2,26.0] | [4.3,10.1] |  |
| 2002/2003 (capitals) | Weighted Prevalence | **23.5** | **8.4** | **56.0** | **9.0** | **3.0** | **100** |
|  | [95% CI] | [16.4,32.5] | [4.9,14.1] | [46.3,65.3] | [6.0,13.5] | [1.7,5.3] |  |
| 2002/2003 (all sample) | Weighted Prevalence | **24.7** | **11.3** | **52.8** | **8.9** | **2.4** | **100** |
|  | [95% CI] | [22.3,27.2] | [9.6,13.1] | [49.9,55.7] | [7.7,10.2] | [1.9,3.0] |  |
| 2010 (capitals) | Weighted Prevalence | **21.5** | **2.9** | **48.2** | **22.9** | **4.6** | **100** |
|  | [95% CI] | [13.1,33.1] | [1.7,4.8] | [39.8,56.7] | [18.7,27.7] | [4.0,5.2] |  |
| 2010 (all sample) | Weighted Prevalence | **26.1** | **3.2** | **39.2** | **24** | **7.5** | **100** |
|  | [95% CI] | [22.1,30.4] | [2.4,4.3] | [34.8,43.8] | [21.4,26.8] | [5.7,9.9] |  |
| 2022/2023 (capitals) | Weighted Prevalence | **32.8** | **5.7** | **44.8** | **14.1** | **2.6** | **100** |
|  | [95% CI] | [29.0,36.7] | [4.9,6.5] | [39.8,49.9] | [12.2,16.3] | [1.9,3.7] |  |
| 2022/2023 (all sample) | Weighted Prevalence | **39.9** | **4.1** | **40** | **12.4** | **3.5** | **100** |
|  | [95% CI] | [35.7,44.3] | [2.9,5.8] | [36.7,43.4] | [9.9,15.3] | [1.8,6.8] |  |
| Older adults 50-74 years | | | | | | | |
| 1986 (capitals) | Weighted Prevalence | **8.2** | **12.0** | **42.2** | **23.9** | **13.7** | **100** |
|  | [95% CI] | [5.3,12.5] | [7.9,18.0] | [31.7,53.3] | [20.4,27.7] | [8.2,22.1] |  |
| 2002/2003 (capitals) | Weighted Prevalence | **21.9** | **12.4** | **53.8** | **10.7** | **1.2** | **100** |
|  | [95% CI] | [10.9,39.0] | [6.0,23.8] | [38.1,68.8] | [5.3,20.2] | [0.3,5.3] |  |
| 2002/2003 (all sample) | Weighted Prevalence | **18.9** | **8.6** | **56.5** | **11.4** | **4.7** | **100** |
|  | [95% CI] | [16.2,21.8] | [6.8,10.7] | [52.9,60.0] | [9.6,13.5] | [3.5,6.2] |  |
| 2010 (capitals) | Weighted Prevalence | **25.6** | **2.7** | **36.9** | **26.5** | **8.4** | **100** |
|  | [95% CI] | [19.3,33.3] | [1.4,4.8] | [32.0,42.0] | [20.6,33.4] | [6.3,11.1] |  |
| 2010 (all sample) | Weighted Prevalence | **31.3** | **1.9** | **33.1** | **26.8** | **7** | **100** |
|  | [95% CI] | [26.2,36.8] | [1.0,3.4] | [27.9,38.7] | [21.4,32.9] | [5.2,9.2] |  |
| 2022/2023 (capitals) | Weighted Prevalence | **31.8** | **5.5** | **41.1** | **17.2** | **4.4** | **100** |
|  | [95% CI] | [28.1,35.8] | [4.2,7.0] | [35.7,46.7] | [14.8,20.0] | [3.0,6.3] |  |
| 2022/2023 (all sample) | Weighted Prevalence | **32.7** | **5.3** | **38** | **19.5** | **4.5** | **100** |
|  | [95% CI] | [28.8,36.8] | [3.9,7.3] | [34.7,41.5] | [16.4,23.0] | [2.9,6.8] |  |

Table S3 – Weighted prevalence of Clinical Attachment Loss categories by survey year and age group in Brazil.

| Survey Year |  | 0-3 mm | 4-5 mm | 6-8 mm | 9-11 mm | >12mm | Total |
| --- | --- | --- | --- | --- | --- | --- | --- |
| Adults 35-44 years | | | | | | | |
| 2002/2003 (capitals) | Weighted Prevalence | **66.5** | **20.6** | **8.8** | **2.9** | **1.2** | **100** |
|  | [95% CI] | [58.6,73.5] | [15.9,26.2] | [6.6,11.7] | [1.8,4.7] | [0.5,3.0] |  |
| 2002/2003 (all sample) | Weighted Prevalence | **66.6** | **21.0** | **8.8** | **2.5** | **1.2** | **100** |
|  | [95% CI] | [64.0,69.0] | [19.4,22.7] | [7.9,9.7] | [2.1,2.9] | [1.0,1.6] |  |
| 2010 (capitals) | Weighted Prevalence | **68.9** | **21.8** | **6.4** | **1.8** | **1.1** | **100** |
|  | [95% CI] | [55.2,80.0] | [14.6,31.2] | [4.2,9.5] | [1.0,3.6] | [0.5,2.2] |  |
| 2010 (all sample) | Weighted Prevalence | **70.9** | **20.3** | **6.7** | **1.5** | **0.5** | **100** |
|  | [95% CI] | [65.2,76.1] | [16.8,24.4] | [5.1,8.8] | [0.9,2.6] | [0.2,1.0] |  |
| 2022/2023 (capitals) | Weighted Prevalence | **84.7** | **12.5** | **2.1** | **0.4** | **0.3** | **100** |
|  | [95% CI] | [83.0,86.2] | [10.8,14.4] | [1.7,2.6] | [0.2,0.6] | [0.2,0.7] |  |
| 2022/2023 (all sample) | Weighted Prevalence | **81.8** | **14.8** | **2.4** | **0.7** | **0.1** | **100** |
|  | [95% CI] | [78.3,84.9] | [11.7,18.7] | [1.6,3.6] | [0.4,1.5] | [0.1,0.3] |  |
| Older adults 65-74 years | | | | | | | |
| 2002/2003 (capitals) | Weighted Prevalence | **44.1** | **36** | **14** | **4.3** | **1.6** | **100** |
|  | [95% CI] | [32.3,56.6] | [25.9,47.6] | [6.3,28.3] | [2.5,7.4] | [0.5,4.9] |  |
| 2002/2003 (all sample) | Weighted Prevalence | **53.1** | **24.8** | **13.5** | **5.7** | **2.8** | **100** |
|  | [95% CI] | [49.8,56.5] | [22.7,27.1] | [11.9,15.3] | [4.6,7.0] | [2.1,3.8] |  |
| 2010 (capitals) | Weighted Prevalence | **52.9** | **31.6** | **11.3** | **2.3** | **2** | **100** |
|  | [95% CI] | [41.1,64.3] | [22.7,42.0] | [8.6,14.8] | [1.3,3.9] | [1.0,3.9] |  |
| 2010 (all sample) | Weighted Prevalence | **62.2** | **24.0** | **8.8** | **3.5** | **1.5** | **100** |
|  | [95% CI] | [54.2,69.6] | [18.6,30.4] | [6.5,11.9] | [1.7,6.9] | [0.7,3.0] |  |
| 2022/2023 (capitals) | Weighted Prevalence | **62.3** | **27.5** | **8.2** | **1.6** | **0.4** | **100** |
|  | [95% CI] | [55.5,68.6] | [23.5,31.8] | [5.9,11.4] | [0.9,2.8] | [0.1,1.0] |  |
| 2022/2023 (all sample) | Weighted Prevalence | **61.2** | **27.2** | **9.0** | **2.3** | **0.3** | **100** |
|  | [95% CI] | [56.5,65.7] | [24.2,30.4] | [7.0,11.6] | [1.3,3.8] | [0.1,0.6] |  |

Table S4 – Weighted prevalence of pocket depth ≥4mm (Community Periodontal Index scores 4+5) according to income by age group and survey year in representative samples of Brazilians.

|  |  | 2002/2003 | | | 2010 | | | 2022/2023 | | |
| --- | --- | --- | --- | --- | --- | --- | --- | --- | --- | --- |
|  |  | % | [95% CI] | P-value | % | [95% CI] | P-value | % | [95% CI] | P-value |
| Young 15-19 years | | | | | | | | | | |
| Total |  | ***1.3*** | [1.1,1.7] |  | ***10.1*** | [7.7,13.1] |  | ***4.7*** | [3.4,6.6] |  |
| Equivalent Household Income | <=1/2MW | **1.8** | [1.2,2.6] | <0.01 | **15.2** | [9.4,23.6] | <0.01 | **3.6** | [2.0,6.3] | 0.22 |
|  | 1/2-1MW | **1.6** | [1.3,2.1] |  | **12.3** | [9.0,16.6] |  | **5.2** | [2.9,9.3] |  |
|  | 1-2MW | **0.9** | [0.6,1.3] |  | **8.3** | [5.1,13.3] |  | **3.8** | [2.2,6.4] |  |
|  | =>2mw | **1.0** | [0.6,1.4] |  | **3.8** | [1.7,8.3] |  | **1.5** | [0.6,3.5] |  |
| Total Household Income | <3 MW | **1.6** | [1.2,2.0] | 0.01 | **11.8** | [8.9,15.4] | 0.11 | **4.4** | [2.9,6.4] | 0.23 |
|  | 3-5 MW | **0.7** | [0.4,1.2] |  | **6.7** | [3.7,11.9] |  | **3.0** | [1.1,7.8] |  |
|  | =>5 MW | **1.2** | [0.8,1.9] |  | **6.1** | [1.2,25.1] |  | **1.2** | [0.4,3.3] |  |
| Adults 35-44 years | | | | | | | | | | |
| Total |  | ***11.3*** | [9.8,13.0] |  | ***31.5*** | [28.3,34.9] |  | ***16.3*** | [13.6,19.4] |  |
| Equivalent Household Income | <=1/2MW | **13.2** | [11.0,15.6] | <0.01 | **41.7** | [35.2,48.5] | <0.01 | **14.3** | [9.7,20.6] | 0.33 |
|  | 1/2-1MW | **12.3** | [10.3,14.6] |  | **34.9** | [29.3,41.0] |  | **17.6** | [13.0,23.4] |  |
|  | 1-2MW | **11.3** | [9.3,13.7] |  | **30.6** | [26.5,35.0] |  | **18.5** | [13.8,24.4] |  |
|  | =>2mw | **8.6** | [6.8,10.8] |  | **22.6** | [18.6,27.1] |  | **11.4** | [6.4,19.5] |  |
| Total Household Income | <3 MW | **12.4** | [10.7,14.3] | <0.01 | **34.9** | [30.8,39.1] | <0.01 | **16.6** | [13.7,19.9] | 0.91 |
|  | 3-5 MW | **11.1** | [8.9,13.7] |  | **27.2** | [22.7,32.1] |  | **15.7** | [10.8,22.2] |  |
|  | =>5 MW | **8.2** | [6.4,10.4] |  | **9.6** | [5.1,17.4] |  | **14.9** | [6.9,29.3] |  |
| Older adults 50-74 years | | | | | | | | | | |
| Total |  | ***16.1*** | [13.6,18.9] |  | ***33.7*** | [27.6,40.3] |  | ***23.8*** | [20.4,27.5] |  |
| Equivalent Household Income | <=1/2MW | **20.8** | [15.7,27.2] | 0.10 | **30.7** | [11.7,59.6] | 0.49 | **28.1** | [16.9,43.0] | 0.12 |
|  | 1/2-1MW | **17.1** | [13.7,21.1] |  | **37.0** | [25.9,49.8] |  | **28.5** | [22.2,35.6] |  |
|  | 1-2MW | **15.2** | [11.8,19.3] |  | **36.5** | [29.2,44.4] |  | **18.9** | [14.0,25.1] |  |
|  | =>2mw | **13.2** | [9.6,17.8] |  | **28.5** | [22.0,35.9] |  | **27.8** | [19.5,38.1] |  |
| Total Household Income | <3 MW | **17.0** | [14.0,20.5] | 0.31 | **36.6** | [28.5,45.6] | 0.20 | **23.9** | [20.2,28.2] | 0.94 |
|  | 3-5 MW | **15.5** | [11.1,21.0] |  | **28.3** | [20.8,37.3] |  | **25.2** | [17.8,34.4] |  |
|  | =>5 MW | **12.7** | [8.6,18.2] |  | **30.1** | [20.5,41.9] |  | **26.0** | [13.8,43.6] |  |

Table S5 – Weighted prevalence of Clinical Attachment Loss ≥4mm according to income by age group and survey year in representative samples of Brazilians.

|  |  | 2002/2003 | | | 2010 | | | 2022/2023 | | |
| --- | --- | --- | --- | --- | --- | --- | --- | --- | --- | --- |
|  |  | % | [95% CI] | P-value | % | [95% CI] | P-value | % | [95% CI] | P-value |
| Adults 35-44 years | | | | | | | | | | |
| Equivalent Household Income | <=1/2MW | **37.3** | [34.1,40.6] | <0.01 | **35.3** | [27.0,44.5] | 0.08 | **20.3** | [14.8,27.3] | 0.23 |
|  | 1/2-1MW | **35.8** | [32.4,39.4] |  | **32.1** | [25.0,40.2] |  | **22.6** | [15.6,31.6] |  |
|  | 1-2MW | **32.8** | [29.9,35.7] |  | **26.5** | [20.3,33.9] |  | **16.4** | [12.6,21.0] |  |
|  | =>2mw | **28.0** | [24.8,31.5] |  | **24.6** | [19.1,31.0] |  | **15.7** | [10.1,23.6] |  |
| Older adults 65-74 years | | | | | | | | | | |
| Equivalent Household Income | <=1/2MW | **47.2** | [39.3,55.2] | 0.59 | **29.7** | [16.7,47.2] | 0.41 | **48.4** | [34.4,62.6] | 0.17 |
|  | 1/2-1MW | **48.2** | [43.5,53.0] |  | **34.3** | [25.5,44.3] |  | **45.1** | [37.5,53.0] |  |
|  | 1-2MW | **47.6** | [42.8,52.4] |  | **41.6** | [32.9,50.8] |  | **36.8** | [30.3,43.7] |  |
|  | =>2mw | **43.7** | [38.2,49.3] |  | **35.7** | [24.6,48.6] |  | **36.3** | [27.0,46.7] |  |

Table S6 – Sensitivity analysis of standardised populations.

| Survey Year | Prevalence 1 -  unweighted and not standardised | Prevalence 2 -  unweighted and age-sex-standardised by sample population within age group. | Prevalence 3 -  weighted and unadjusted | Prevalence 4 -  weighted sex-standardised | Prevalence 5 -  weighted age standardised |
| --- | --- | --- | --- | --- | --- |
| Community Periodontal Index – Pocket Depth ≥4mm | | | | | |
| Young 15-19 years | | | | | |
| 1986 | 7.8% | 7.7% | 7.8% | 7.7% | 7.7% |
| 2002/2003 | 1.2% | 1.2% | 1.2% | 1.2% | 1.1% |
| 2010 | 8.0% | 8.0% | 8.9% | 8.7% | 8.8% |
| 2022/2023 | 4.5% | 4.6% | 5.5% | 5.5% | 5.9% |
| Adults 35-44 years | | | | | |
| 1986 | 27.9% | 28.5% | 27.9% | 28.8% | 28.5% |
| 2002/2003 | 12.0% | 11.9% | 12.0% | 13.5% | 12.4% |
| 2010 | 27.9% | 27.6% | 27.5% | 28.2% | 27.7% |
| 2022/2023 | 16.8% | 16.7% | 16.8% | 17.1% | 16.8% |
| Older adults 50-74 years | | | | | |
| 1986 | 37.6% | 4.4% | 37.6% | 36.9% | 19.0% |
| 2002/2003 | 11.8% | 9.9% | 11.8% | 11.6% | 5.9% |
| 2010 | 32.5% | 28.6% | 34.9% | 35.9% | 17.3% |
| 2022/2023 | 21.0% | 18.6% | 21.6% | 22.5% | 10.9% |
| Community Periodontal Index – Clinical Attachment Loss ≥4mm | | | | | |
| Adults 35-44 years | | | | | |
| 2002/2003 | 33.4% | 33.7% | 33.4% | 35.0% | 34.2% |
| 2010 | 26.9% | 26.8% | 29.1% | 29.7% | 29.9% |
| 2022/2023 | 16.5% | 16.3% | 18.2% | 18.7% | 18.1% |
| Older adults 65-74 years | | | | | |
| 2002/2003 | 46.9% | 46.0% | 46.9% | 46.9% | 46.6% |
| 2010 | 40.4% | 40.4% | 37.8% | 38.9% | 39.1% |
| 2022/2023 | 38.3% | 38.6% | 38.8% | 39.8% | 38.7% |

Figure S1 – Trends in prevalence and 95% confidence intervals (for survey years) of Community Periodontal Index scores 4 and 5 (periodontal probing depth ≥4mm) adjusted by sex, age and dental visits in three income groups in Brazilian state capitals between 1986 and 2023 (Test of differences of trends among income groups: a) 15-19 year p=0.21; b) 35-44 years p<0.01; c) 50-74 years p=0.01).


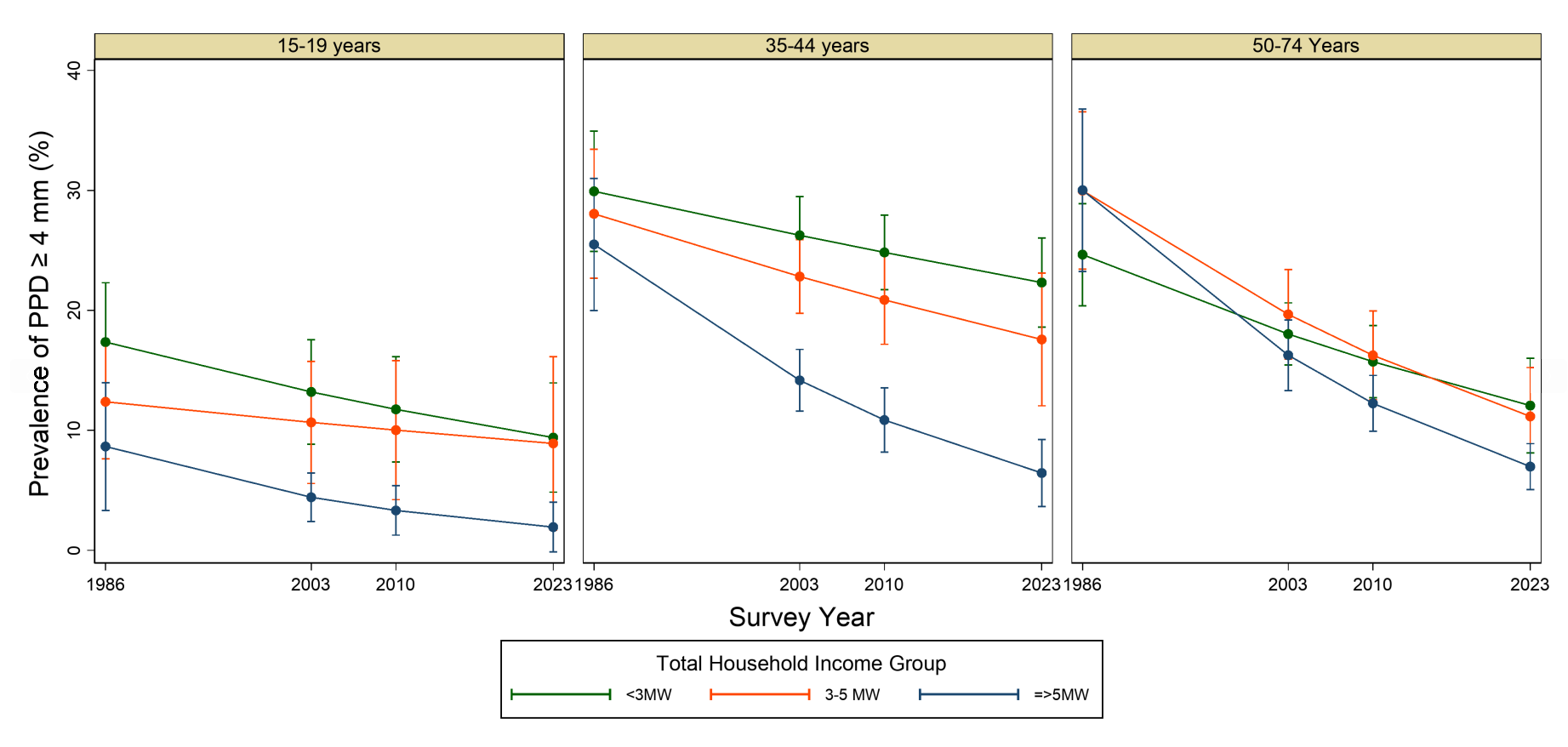

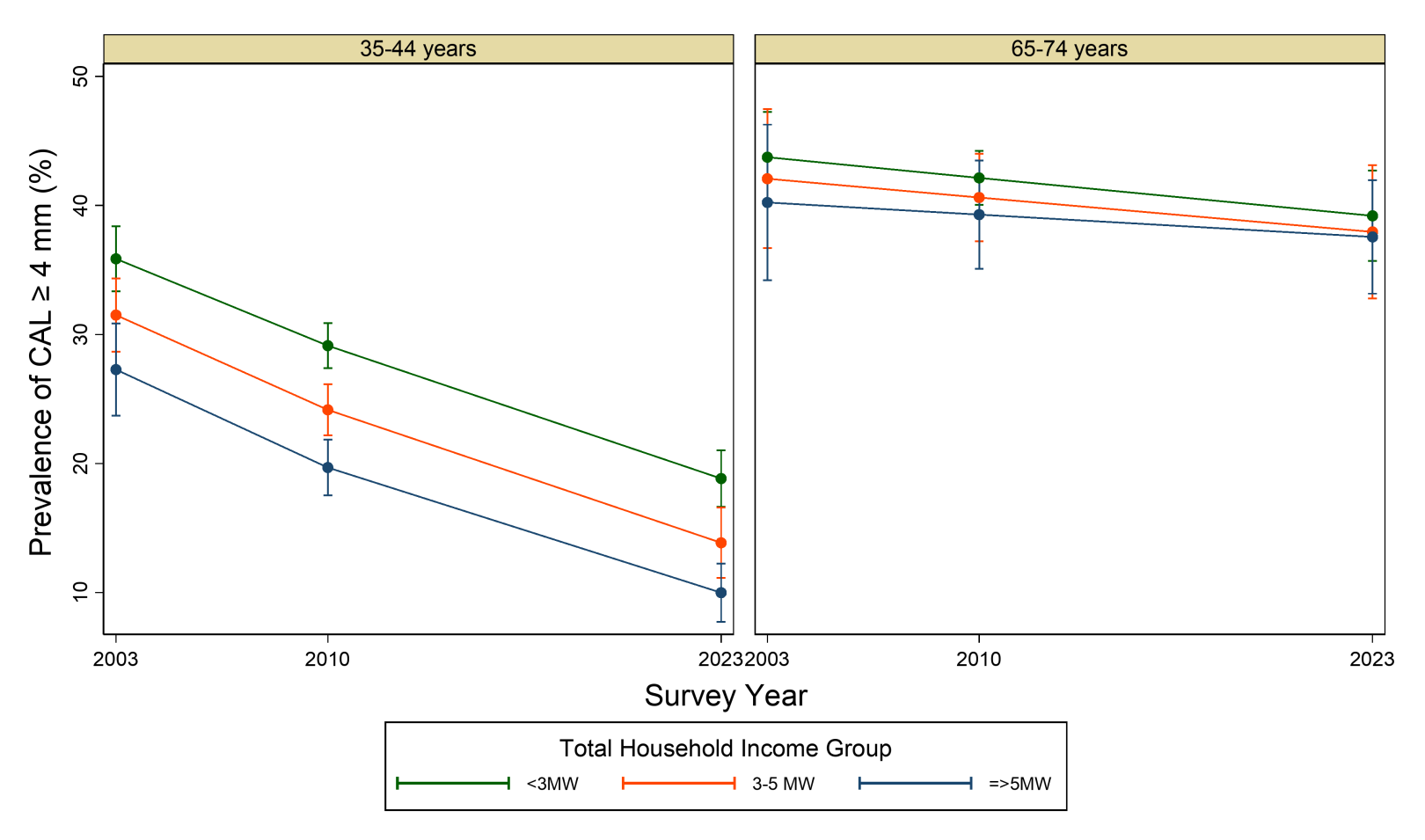


Figure S2 – Trends in prevalence and 95% confidence intervals (for survey years) of clinical attachment loss ≥4mm adjusted by sex, age and dental visits in three income groups in Brazil between 2003 and 2023.(Test of differences of trends among income groups: a) 35-44 years p=0.04; b) 65-74 years p=0.96).
